# Sociodemographic traits as early indicators of AD, FTD, and VaD up to 10 years before diagnosis

**DOI:** 10.1002/alz.70616

**Published:** 2025-09-24

**Authors:** Ave Kivisild, Iina Rinnankoski, Mikko Aaltonen, Kalle Aho, Sami Heikkinen, Adolfina Lehtonen, Laura Leppänen, Helmi Soppela, Laura Tervonen, Kaijus Ervasti, Päivi Hartikainen, Annakaisa Haapasalo, Kasper Katisko, Johanna Krüger, Eino Solje

**Affiliations:** ^1^ Institute of Clinical Medicine—Neurology University of Eastern Finland Kuopio Finland; ^2^ Research Unit of Clinical Medicine Neurology University of Oulu Oulu Finland; ^3^ Law School University of Eastern Finland Joensuu Finland; ^4^ Medical Research Center Oulu University Hospital Oulu Finland; ^5^ Neurocenter Neurology Oulu University Hospital Oulu Finland; ^6^ Neuro Center—Neurology Kuopio University Hospital Kuopio Finland; ^7^ A.I. Virtanen Institute for Molecular Sciences University of Eastern Finland Kuopio Finland

**Keywords:** alzheimer's disease, education, frontotemporal dementia, marital status, sociodemographics, vascular dementia

## Abstract

**INTRODUCTION:**

We aimed to investigate early differences in sociodemographic factors before the onset of Alzheimer's disease (AD), frontotemporal dementia (FTD), vascular dementia (VaD), and mixed dementia (AD + VaD).

**METHODS:**

Lifetime sociodemographic factors were collected from Statistics Finland for 1238 AD, 274 FTD, 343 VaD, and 402 AD + VaD patients with a diagnosis and visit at Kuopio and Oulu University Hospitals between January 2010 and December 2021. Comparisons were performed between dementia groups and matched controls.

**RESULTS:**

All patient groups showed decreased employment status compared to controls already 10 years prior to diagnosis. In particular, individuals with early‐onset FTD (EOFTD; 66.9% vs. 77.6%, *p* < 0.01) and early‐onset VaD (EOVaD; 49.0% vs. 76.5%, *p* < 0.001) had significantly lower employment rates than controls. Similarly, 10 years prior to diagnosis the proportion of married individuals was lower in the VaD (60.1% vs. 65.2%, *p* < 0.05) and EOVaD (50.0% vs. 61.6%, *p* < 0.05) groups versus controls, while single status was more common in early‐onset AD (EOAD; 23.2% vs. 17.0%, *p* < 0.01) versus controls. Patients with VaD and AD + VaD had lower levels of education than controls: basic education only in 51.9% of VaD (vs. 45.0%, *p* < 0.05) and 65.7% of AD + VaD (vs. 60.2%, *p* < 0.05).

**DISCUSSION:**

Our findings may aid in the early recognition or potential risk factor evaluation for different types of dementia. Screening cognitive symptoms in individuals with unexplained long‐term unemployment may help detect prodromal dementia.

**Highlights:**

- Employment rates were already reduced 10 years before the diagnosis of Alzheimer's disease, frontotemporal dementia, and vascular dementia.
- The association between education level and dementia risk appears to be subtype specific.
- Lower employment may serve as an early “social marker” of subtle cognitive decline.
- Social markers could help inform models predicting progression to cognitive impairment.

## 1 BACKGROUND

Increased life expectancy has generated new medical and societal challenges due to the globally increasing number of people suffering from age‐related, chronic diseases like Alzheimer's disease (AD), vascular dementia (VaD), and other major cognitive disorders.^1^ Previous studies have shown that all‐cause dementia is associated with certain sociodemographic factors, including the level of education, socioeconomic status, and social isolation.^2^ The identification of these sociodemographic traits is important, because it can lead to a better understanding of the first clinical signs and potential modifiable risk factors for dementia. This would improve health‐care services and diagnostic pathways, reduce the societal economic burden caused by these diseases, and provide new pathophysiological insight and preventive measures.

So far, only a few previous studies have investigated the relationship between employment status and neurodegenerative diseases. Most of these studies are qualitative reports based on interviews with a small number of patients.^3^, ^4^, ^5^ Only one small quantitative study has previously been published demonstrating the time course of job loss among 220 patients with early‐onset dementia (EOD).^6^ To the best of our knowledge, no larger quantitative follow‐up studies assessing the association of employment status with EOD or late‐onset dementia (LOD) have been conducted.

Lower level of education and a non‐marital status are known risk factors for all‐cause dementia.^1^, ^2^, ^7^, ^8^, ^9^ A large Swedish population‐based prospective study focusing only on a single sociodemographic factor estimated that living alone as non‐married may increase the risk for EOD and LOD.^1^ There is currently a lack of large, comprehensive longitudinal studies that systematically assess sociodemographic factors in different dementia subgroups.

The aim of the present study was to evaluate in a large and comprehensive dataset, the longitudinal differences in various sociodemographic traits, either as early signs or potential risk factors, in patients with AD, frontotemporal dementia (FTD), and VaD compared to age‐, sex‐, and region‐matched individuals without a neurodegenerative disease (including the exclusion of non‐cognitive neurodegenerative diseases).

## 2 METHODS

### 2.1 Standard protocol approvals, registrations, and patient consents

This study was approved (THL/2841/14.02.00/2022) by the Finnish Social and Health Data Permit Authority Findata (www.findata.fi/en). Finnish legislation (Act on the Secondary Use of Health and Social Data, 552/2019) does not mandate consent for retrospective studies without patient contact, so no ethical committee evaluation was needed. This study is part of DEGE‐RWD research project (NCT06209515), coordinated by Neurocenter Finland.

### 2.2 Study population

We conducted this study in the provinces of Northern Ostrobothnia (37,149 km^2^) located in Northern Finland, and in Northern Savonia (20,367 km^2^) in Eastern Finland. Kuopio University Hospital (KUH) and Oulu University Hospital (OUH) represent tertiary‐level neurological clinics in these areas and are the primary regional referral centers for all citizens < 65 years of age with neurodegenerative disease–related cognitive complaints. Thus, the EOD diagnostics are performed exclusively within these two centers. EOD defines all neurodegenerative and related disorders causing dementia with onset < 65 years of age.^10^ LODs are being diagnosed also outside these hospitals. Therefore, for LOD the coverage is not complete. Each patient included in this study received their initial diagnosis in these two specialized memory centers (KUH or OUH), by a neurologist specialized in neurodegenerative diseases.

All consecutive patients referred to OUH and KUH neurology outpatient clinics with a progressive neurodegenerative disease during 2010 through 2021 were identified from KUH and OUH data registry by International Classification of Diseases codes. To maximize sensitivity, we used an extensive list of diagnostic codes (156 codes) related to cognitive disorders and neurodegenerative diseases to ensure inclusion of all relevant patients, including those with suspected conditions. For research purposes, we further conducted a manual retrospective re‐evaluation of all automatically identified cases (*n* = 12,490) by reviewing all relevant health‐care records to ensure they met the relevant diagnostic criteria. In cases in which there was diagnostic uncertainty, patients were followed‐up clinically to confirm or rule out the diagnosis. If the follow‐up was still ongoing or the diagnosis remained unclear, the patient was excluded from the study. Eventually, after the retrospective re‐evaluation, 2257 individuals were confirmed to have a clinical diagnosis of AD, FTD, or VaD. The eventual study cohort included 1238 AD, 274 FTD (221 behavioral variant FTD [bvFTD] and 53 primary progressive aphasia), 343 VaD, and 402 AD + VaD patients. The AD group included only typical AD phenotype; patients with atypical AD presentations were excluded. Medical records (including all the health data after the diagnosis) were uniformly reviewed using established diagnostic criteria,^11^, ^12^, ^13^, ^14^ and each diagnosis was confirmed for data validation. Approximately 60% of the patients with AD were confirmed with cerebrospinal fluid (CSF) amyloid and tau markers.^15^

In Finland, each citizen has a personal identity code (Finnish social security number), which can be linked to comprehensive national registry data for each subject. First, the clinical data collected were sent to the Finnish Social and Health Data Permit Authority for combination. Based on the Finnish social security number, these individuals were linked to Statistics Finland national register data on annual (1987–2020) reports of education, employment, and marital status, and made available to the research group in pseudonymized form in a Statistics Finland secure remote environment. Education level was assessed based on the most recent entry (typically from the year of diagnosis), to capture all formal education completed before diagnosis. Co‐habiting adults were included to the group of “married,” excluding the adult children living with the study participant. In Finland, cohabitation can legally be equated with marriage. For each study case, 10 control cases without a diagnosis of neurodegenerative disease (randomly sampled by Digital and Population Data Services Agency), matched with age, sex, and geographical area, were sought. We used cumulative density sampling (survivor sampling), in which the controls were required to remain without a diagnosis of dementia until the end of 2021 and be alive on June 30 of the diagnosis year of the case.

### 2.3 Statistical analyses

In each diagnosis group, the differences between the cases and their matched controls were examined using cross‐tabulations and Pearson chi‐squared tests. For variables with multiple categories, we used both omnibus tests and also examined the same associations using binary versions of the same outcomes (e.g., employed vs. the rest). We examined sociodemographic outcomes at three time points: 10 and 5 years before the diagnosis, and at the year of the diagnosis. Given that 10 age‐ and sex‐matched control cases were not available for all cases from the same geographic region, we additionally tested the robustness of our results using conditional logistic regression models suitable for case–control designs in which the number of controls varies between cases. These models gave substantively similar results to the primary analyses. The results are presented using the odds ratio metric, as detailed in Table S1 in supporting information.

**RESEARCH IN CONTEXT**

1. **Systematic review**: The authors reviewed existing literature on dementia and sociodemographic factors using PubMed. While prior studies link dementia risk to education, marital status, and employment, few have explored how these factors change longitudinally before diagnosis, especially in early‐onset dementia.
2. **Interpretation**: Our results demonstrate that minor cognitive symptoms may cause sociodemographic changes, especially decreased employment status (as a “social marker”) before clear cognitive symptoms occur. Moreover, the association between education level and dementia risk appears to be subtype specific, highlighting the importance of tailored approaches to prevention and care.
3. **Future directions**: Understanding prediagnostic sociodemographic patterns could improve early detection. Markers such as long‐term unemployment or unmarried status may help identify individuals at elevated risk and support timely intervention.

**FIGURE 1:**
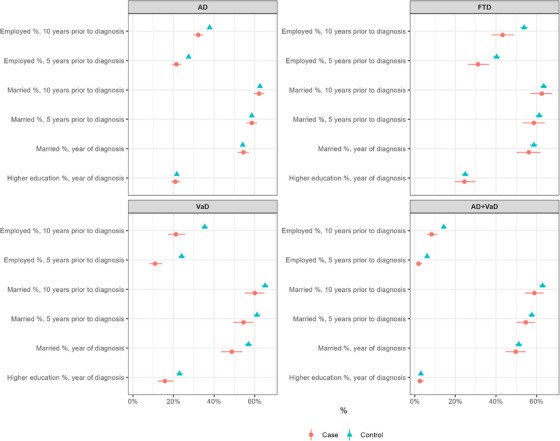
Comparisons of main sociodemographic factors between the dementia groups and the control groups with their 95% confidence intervals. AD, Alzheimer's disease; FTD, frontotemporal dementia; VaD, vascular dementia

**FIGURE 2:**
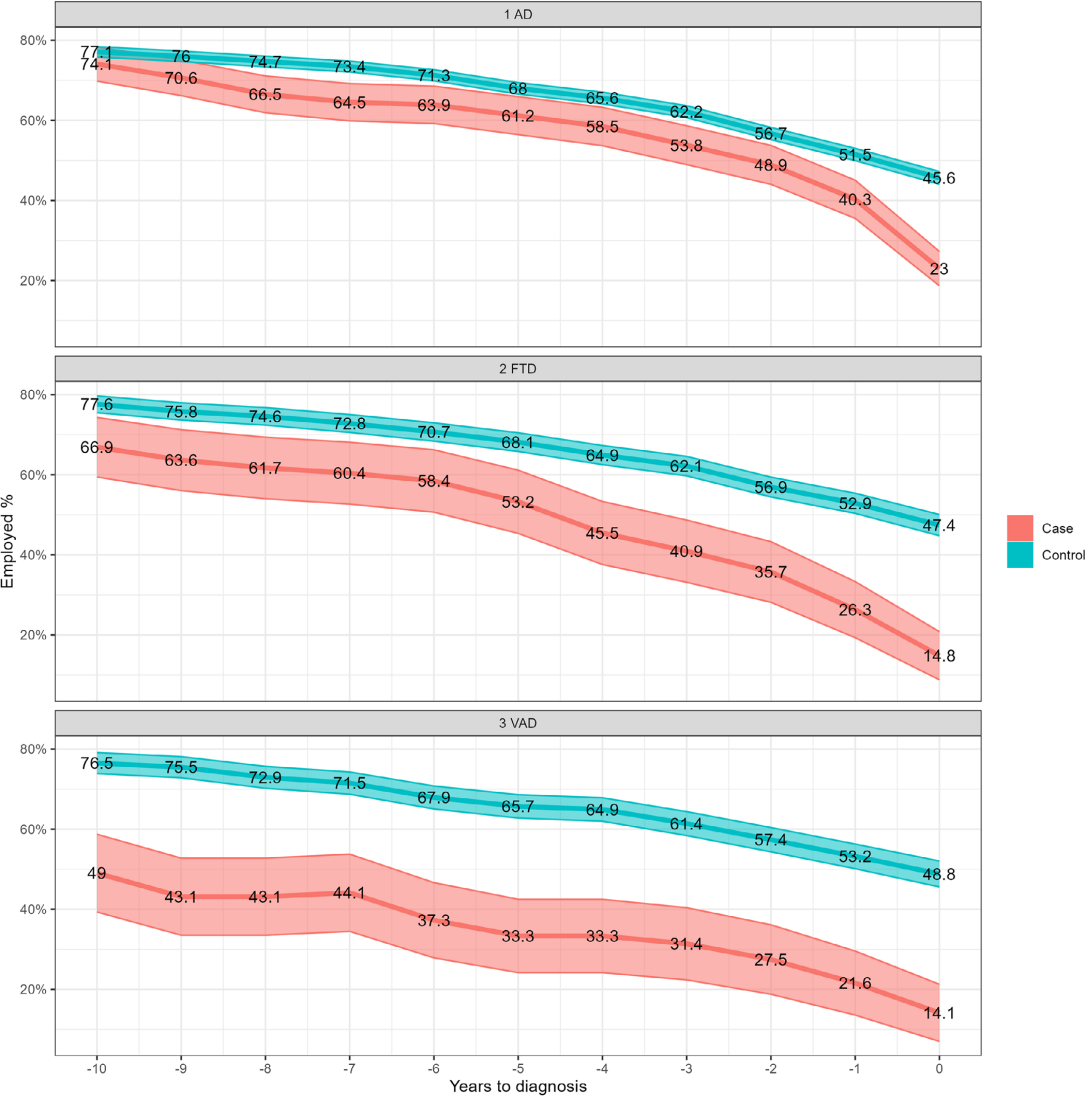
Employment status of early‐onset dementia patients, including Alzheimer's disease, frontotemporal, and vascular dementia. Predicted probabilities and their 95% confidence intervals calculated from logistic regression models with cluster‐robust standard errors. AD, Alzheimer's disease; FTD, frontotemporal dementia; VaD, vascular dementia

Although the descriptive analyses in Table 1 and Figure 1 are presented at fixed time points (10 and 5 years before diagnosis and at the year of diagnosis), the dataset is longitudinal in structure. Each individual has repeated annual data points, allowing us to model temporal trends. In particular, Figure 2 was generated using a logistic regression model with a time × group interaction term, capturing the group‐specific evolution of employment probability over the 10 year period. These models used clustered standard errors to account for within‐subject correlation. All analyses were conducted using Stata 18 software.

**TABLE 1:** Characteristics of the study cohort and comparisons of sociodemographic factors between the dementia and the control groups.

	AD (*N* = 1238)/controls (*N *= 10 789)	FTD (*N* = 274)/controls (*N* = 2746)	VaD (*N* = 343)/ controls (*N* = 3083)	AD + VaD (*N* = 402)/controls (*N* = 3328)
Sex, male (%)	725 (58.6)	138 (50.4)	138 (40.2)	195 (48.5)
Age at diagnosis, mean (SD)	71.8 (9.8)	65.8 (8.9)	71.9 (9.9)	77.9 (6.3)
Mean time (years) from symptoms to diagnosis (SD)	2.6 (2.2)	3.3 (3.5)	2.5 (2.5)	2.5 (2.2)
**10 years before diagnosis**				
**Employment status % (95% CI)**				
Employed	32.2 (29.7–34.7)/37.8 (36.9–38.7)***	43.3 (37.8–48.9)/53.9 (52.1–55.7)***	21.2 (17.3–25.6)/35.4 (33.8–37.0)***	8.3 (6.0–11.2)/14.3 (13.2–15.4)**
Retired (disability pension included)	59.2 (56.5–61.8)/51.6 (50.7–52.5)***	37.7 (32.4; 43.3)/32.9 (31.2; 34.6)	67.7 (62.9; 72.2)/54.7 (53.0; 56.4)***	86.0 (82.4; 89.0)/78.1 (76.7; 79.4)***
Other, not employed	8.6 (7.2–10.2)/10.7 (10.1–11.2)	19.0 (15.0–23.8)/13.2 (12.1–14,5)***	11.1 (8.3–14.7)/10.0 (9.0–11.0)	5.7 (3.9–8.3)/7.7 (6.8–8.6)
**Marital status (%)**				
Single	12.6 (10.9–14.5) / 12.4 (11.8–13.0)	13.7 (10.3–18.1)/15.3 (14.1–16.7)	14.3 (11.1–18.2)/13.1 (12.0–14.3)	11.9 (9.2–15.3)/10.8 (9.8–11.8)
Married	62.1 (59.5–64.7)/62.6 (61.7–63.5)	62.6 (57.1–67.9)/63.6 (61.2–65.3)	60.1 (55.0–64.9)/65.2 (63.5–66.7)*	58.9 (54.2–63.5)/63.0 (61.4–64.5)
Divorced/separated	11.2 (9.6–13.0)/13.5 (12.8–14.1)*	17.7 (13.8–22.4)/15.6 (14.3–16.9)	13.0 (9.9–16.7)/13.2 (12.1–14.4)	11.2 (8.6–14.5)/11.0 (9.9–12.0)
Widowed	14.1 (12.3–16.1)/11.5 (11.0–12.1)**	5.9 (3.7–9.2)/5.4 (4.8–6.4)	12.7 (9.7–16.4)/8.5 (7.6–9.5)**	17.9 (14.6–21.8)/ 15.3 (14.2–16.5)
**5 years before diagnosis**				
**Employment status % (95% CI)**				
Employed	21.4 (19.3–23.7)/27.5 (26.7–28.3)***	31.1 (26.2–36.6)/40.3 (38.6–42.1)**	10.9 (8.1–14.4)/24.0 (22.6–25.5)***	1.8 (0.9–3.6)/6.1 (5.3–6.9)***
Retired (disability pension included)	71.2 (68.7–73.6)/65.3 (64.4–66.2)***	54.4 (48.8–60.0)/49.6 (47.8–51.4)	81.2 (77.0–84.8)/ 68.6 (67.0–70.1)***	95.6 (93.3–97.2)/90.1 (89.9–91.8)**
Other, not employed	7.4 (6.1–8.9)/ 7.2 (6.8–7.7)	14.4 (10.9–18.8)/10.1 (9.1–11.3)*	7.9 (5.6–11.1)/7.4 (6.6–8.4)	2.5 (1.4–4.5)/3.0 (2.5–3.6)
**Marital status %**				
Single	12.1 (10.4–13.9)/12.1 (11.5–12.7)	12.7 (9.5–17.0)/14.65 (13.4–16.0)	14.3 (11.1–18.2)/12.8 (11.7–14.0)	11.5 (8.8–14.8)/10.7 (9.7–11.7)
Married	58.6 (55.9–61.2)/58.6 (57.7–59.5)	59.0 (53.1–64.1)/61.3 (59.6–63.0)	54.5 (49.4–59.5)/61.1 (59.6–62.8)*	54.7 (50.0–59.3)/57.7 (56.1–59.3)
Divorced/separated	11.9 (10.3–13.8)/14.2 (13.6–14.9)*	19.3 (15.3–24.2)/16.6 (15.4–18.0)	14.3 (11.1–18.2)/14.2 (13.2–15.4)	11.3 (8.6–14.6)/11.2 (10.2–12.3)
Widowed	17.5 (15.5–19.6)/15.1 (14.5–15.8)**	9.2 (6.4–13.0)/7.4 (6.5–8.4)	16.9 (13.5–21.1)/11.9 (10.9–13.0)**	22.5 (18.8–26.7) /20.4 (19.1–21.8)
**At the time of diagnosis**				
**Marital status % (95% CI)**				
Single	11.8 (10.1–13.7)/11.8 (11.2–12.4)	12.4 (9.0–16.9)/14.0 (12.7–15.3)	14.0 (10.7–18.1)/12.2 (11.1–13.4)	10.2 (7.6–13.6)/10.6 (9.6–11.7)
Married	54.4 (51.7–57.2)/54.1 (53.2–55.0)	56.2 (50.3–62.0)/58.7 (56.8–60.5)	48.7 (43.4–54.0)/56.9 (55.2–58.7)**	49.8 (44.9–54.6)/51.2 (49.6–53.0)
Divorced/separated	11.6 (9.9–13.5)/14.2 (13.6–14.9)**	19.3 (15.1–24.5)/17.3 (16.0–18.8)	16.6 (13.0–20.9)/14.6 (13.0–15.9)	11.2 (8.5–14.7)/10.9 (9.8–12.0)
Widowed	22.2 (20.0–24.6)/19.9 (19.2–20.7)	12.0 (8.7–16.5)/10.0 (8.9–11.2)	20.7 (16.7– 25.3)/16.3 (15.1–17.7)*	28.9 (24.6–33.5)/27.3 (25.8–28.8)
**Education % (95% CI)**				
Nine‐year basic	48.0 (45.2–50.8)/45.5 (44.5–46.4)	36.1 (30.7–42.0)/37.8 (36.0–39.6)	51.9 (46.6–57.1)/45.0 (43.2–46.7)*	65.7 (60.9–70.2)/60.2 (58.5–61.9)*
Upper secondary	31.2 (28.7–33.8)/33.0 (32.1–33.9)	39.4 (33.8–45.3)/37.4 (35.6–39.2)	32.3 (27.6–37.5)/32.1 (30.5–33.8)	18.9 (15.4–23.0)/24.6 (23.1–26.0)*
Higher	20.8 (18.7–23.2)/21.6 (20.8–22.4)	24.5 (19.7–29.9)/24.9 (23.2–26.5)	15.7 (12.3–20.0)/22.9 (21.5–24.5)**	15.4 (12.2–19.3)/15.3 (14.1–16.5)

*Note*: Table 1 presents percentages with 95% confidence intervals. Each separate box has separate percentages for the patient group and its matched control group. Each separate box (when applicable) also includes *, **, or *** symbols to indicate differences that are statistically significant (* for *p* < 0.05, ** for *p* < 0.01, and *** for *p* < 0.001). Statistical comparisons are made with Pearson chi‐squared tests. All *p* values are calculated between the disease group and its matched healthy controls.

Abbreviations: AD, Alzheimer's disease; CI, confidence interval; FTD, frontotemporal dementia; SD, standard deviation; VaD, vascular dementia.

### 2.4 Data availability

Patient‐level data cannot be shared outside the research group due to data privacy legislation.

## 3 RESULTS

The study cohort and comparisons of sociodemographic factors between the disease groups and the control group are described in Table 1 and Figure 1.

### 3.1 Employment status

First, we evaluated the employment status in the total study cohort including all age groups. There were statistically significant differences in the employment status in all dementia groups compared to age‐ and sex‐matched disease group–specific controls in each examined time period: 10 years before diagnosis, 5 years before the diagnosis, and at the year of receiving the diagnosis (Table 1, Figure 1). For example, FTD patients had significantly lower employment rate already 10 years before diagnosis compared to controls (43.3% vs. 53.9%, *p* < 0.001). Similarly, the employment rate was lower in AD versus controls (32.2 vs. 37.8%, *p* < 0.001) and VaD versus controls (21.2% vs. 35.4%, *p* < 0.001) already 10 years before diagnosis. In the AD + VAD group, 86.0% of the patients were retired already 10 years before the diagnosis, compared to 78.1% in matched controls (*p* < 0.001; Table 1, Figure 1).

Only 7.0% (95% confidence interval [CI] 5.7%–8.6%) of AD patients versus 15.9% (95% CI 15.2%–16.6%) of controls (*p* < 0.001), 7.7% (95% CI 5.0%–11.5%) of FTD patients versus 24.2% (95% CI 22.6%–25.8%) of controls (*p* < 0.001) and 4.0% (95% CI 2.4%–6.8%) of VaD patients versus 15.2% (95% CI 13.9%–16.5%) of controls (*p* < 0.001) were employed at the time of diagnosis. None of the individuals of the AD + VaD group were employed at the time of diagnosis.

In addition, we specifically assessed differences in the employment status prior to the diagnosis in the EOD group patients aged 30 to 65 years (Figure 2). To these analyses 370 early‐onset AD (EOAD), 135 early‐onset FTD (EOFTD), and 92 early‐onset VaD (EOVaD) patients were included with 3699, 1375, and 922 age‐ and sex‐ matched controls, respectively. The early‐onset AD  + VaD group (*N* = 16) was excluded from the analysis. Overall, employment rates were lower in EOD patient groups compared to their matched control groups: 49% (95% CI 39.4%–58.6%) of EOVaD patients versus 76.5% (95% CI 73.8%–79.0%) of controls (*p* < 0.001), 66.9% (95% CI 59.1%–73.9%) of EOFTD patients versus 77.6% (95% CI 75.4%–79.6%) of controls (*p* < 0.01), and 74.1% (95% CI 69.6%–78.1%) of EOAD patients versus 77.1% (95% CI 75.8%–78.4%) of controls (*p* = 0.166) were actively working 10 years before the diagnosis. In the EOFTD group, 18.8% (95% CI 13.4%–25.8%) of patients versus 12.7% (95% CI 11.1%–14.4%) of controls (*p* < 0.05) were unemployed and 14.3% (95% CI 9.6%–20.7%) of patients versus 9.8% (95% CI 8.4%–11.4%) of controls (*p* = 0.077) were retired 10 years before the diagnosis. Only 14.1% (95% CI 8.4%–22.8%) of EOVaD patients versus 48.8% (95% CI 45.6%–52.0%) of controls (*p* < 0.001), 14.8% (95% CI 9.8%–21.9%) of EOFTD patients versus 47.4% (95% CI 44.8%–50.1%) of controls (*p* < 0.001), and 23.0% (95% CI 19.0%–27.5%) of EOAD patients versus 45.7% (95% CI 44.0%–47.2%) of controls (*p* < 0.001) were employed at the time of diagnosis.

### 3.2 Marital status

Statistically significant differences in marital status were observed specifically in the AD and VaD groups compared to controls in several time periods (Table 1, Figure 1). Patients in the VaD group were less often married throughout the follow‐up period compared to controls: 10 years before diagnosis: 60.1% (95% CI 55.0%–64.9%) versus 65.2% (95% CI 63.5%–66.7%), *p* < 0.05; 5 years before diagnosis 54.5% (95% CI 49.4%–59.5%) versus 61.1% (95% CI 59.5%–62.8%), *p* < 0.05; and at the time of diagnosis 48.7% (95% CI 43.4%–54.0%) versus 56.9% (95% CI 55.1%–58.7%), *p* < 0.01. On the other hand, widowed individuals were observed significantly more often specifically in the AD and VaD groups in each time point: for example, at the 10 year time point before diagnosis, 14.1% of AD patients versus 11.5% of controls (*p* < 0.01) and 12.7% of VaD versus 8.5% of controls (*p* < 0.01) were widowed. Divorces were less likely specifically in AD patients compared to controls. For example, 11.2% (95% CI 9.6%–13.0%) of AD patients versus 13.5% (95% CI 12.8%–14.1%) of controls (*p* < 0.05) were divorced 10 years before the diagnosis, and a similar difference was also observed in other time points.

We also examined the differences in marital status in different dementia types separately for males and females. In the total study cohort (all age groups), females diagnosed with VaD were less often married than their female control group, both 5 years prior to diagnosis (38.4%, 95% CI 31.0%–46.4% vs. 51.2%, 95% CI 48.5%–53.8% married, *p* < 0.001) and at the time of diagnosis (31.4%, 95% CI 24.2%–39.6% vs. 43.9%, 95% CI 41.1%–46.6% married, *p* < 0.001). Five years prior to diagnosis, widowed status was also more common among females diagnosed with VaD compared to female controls (32.5%, 95% CI 25.5%–40.3% vs. 22.4%, 95% CI 20.3%–24.7% *p* < 0.01).

In the EOD population, the proportion of unmarried individuals was significantly higher in both the EOVaD and EOAD groups compared to controls, already 10 years prior to diagnosis. Patients with EOVaD were significantly less likely to be married than controls at all assessed time points: 10 years prior to diagnosis 50.0% (95% CI 40.4%–59.6%) versus 61.6% (95% CI 58.5%–64.5%), *p* < 0.05; 5 years prior to diagnosis 46.1% (95% CI 36.7%–55.8%) versus 59.1% (95% CI 56.1%–62.1%), *p* < 0.05; and at the time of diagnosis 40.2% (95% CI 30.7%–50.5%) versus 59.1% (95% CI 55.9%–62.2%), *p* < 0.001.

In the EOAD group, the proportion of single individuals was also significantly higher compared to controls 10 years prior to diagnosis 23.2% (95% CI 19.3%–27.6%) versus 17.0% (95% CI 15.9%–18.2%), *p* < 0.01; 5 years prior to diagnosis 21.9% (95% CI 18.1%–26.1%) versus 16.2% (95% CI 15.1%–17.4%), *p* < 0.01; and at the time of diagnosis 22.7% (95% CI 18.7%–27.2%) versus 15.7% (95% CI 14.5%–16.9%), *p* < 0.001.

In the EOAD group, the proportion of especially single females at the time of diagnosis was significantly higher than among their matched female controls 21.5% (95% CI 16.5%–27.5%) versus 13.3% (95% CI 11.9%–14.7%) *p* < 0.001.

### 3.3 Education

A lower level of education was detected specifically in patients with VaD or AD + VaD: especially VaD, but also AD + VaD patients, were less educated compared to their matched controls (Table 1, Figure 1). Basic education (9 years) was more common in VaD patients 51.9% (95% CI 46.6%–57.1%) compared to controls 45.0% (95% CI 43.2%–46.7%), *p* < 0.05. Similarly, the VaD group had less highly educated subjects 15.7% (95% CI 12.3%–20.0%) than the controls 22.9% (95% 21.5%–24.5%), *p* < 0.01. In addition, the VaD + AD group had more subjects with only 9 years of basic education 65.7% (95% CI 60.9%–70.2%) compared to controls 60.2% (95% CI 58.5%–61.9%), *p* < 0.05.

The level of education did not significantly differ in the separate EOD subgroups compared to controls.

## 4 DISCUSSION

In this extensive study using a validated cohort and comprehensive national registers, we identified a significant association between lower employment rates, observed up to 10 years prior to diagnosis, and the subsequent development of AD, FTD, VaD, and AD + VaD. In the EOD group, decreased employment status and higher rate of unemployment specifically associated with EOFTD and EOVaD patients 10 years before diagnosis. The level of education did not differ in FTD, AD, or in the separately analyzed EOD groups compared to controls. However, AD + VaD and pure VaD patients were less educated compared to controls. Non‐marital status was more common specifically with VaD patients, but also with EOAD patients.

We found that the employment rates were already significantly lower in the dementia groups compared to their matched controls at least 10 years prior to diagnosis of a neurodegenerative disease in all of the examined dementia groups. Assessing only EOD patients, significantly decreased work ability and higher unemployment rate were observed in the EOFTD and EOVaD groups 10 years before the diagnosis. Lower employment rates in dementia groups may represent an early “social marker,” reflecting minimal cognitive dysfunction or functional decline. Alternatively, they could reflect pre‐existing vulnerabilities or social factors associated with increased dementia risk, which is, however, unlikely, because the neurodegenerative process starts generally already decade(s) before the onset of the diagnosis.^16^, ^17^ Different dementia phenotypes may also represent differing reasons for the higher unemployment rate in each time point before dementia diagnosis. For example, lower employment rates in AD and VaD patients were primarily accompanied by higher retirement rates compared to controls, whereas in bvFTD patients, reduced employment was more often associated with non‐retirement–related categories, potentially reflecting factors such as impulsive resignations or other employer‐related causes of unemployment. Our study does not investigate causality. However, the lack of an accurate and early diagnosis may have contributed to unemployment, as the patients were not eligible for a disability pension. In addition, a lack of insight is common in FTD patients, which can lead to patients refusing to undergo clinical investigations and, as the diagnosis is delayed, the patient becomes eventually unemployed.

Almost all subjects in the total study cohort were no longer active in the labor force at the time of diagnosis. Our findings are consistent with previous results showing that most of the patients with dementia leave their jobs shortly after receiving the diagnosis.^6^ On the other hand, many patients with EOD are still working when their early symptoms appear. Therefore, it is crucial that these patients would receive an early diagnosis in a timely manner and the needed support in making the decision whether it is possible for them to keep working after the diagnosis.^3^, ^4^ Mayrhofer et al. revealed that post‐diagnostic information related to employment and legal and financial issues is critically under‐recognized.^18^ In their qualitative study that assessed financial impact of a diagnosis of EOD patients and families, they identified that the primary economic consequences stemmed from the premature loss of income and the reduction or deferral of pension entitlements.^18^ Overall, our findings highlight the importance of allocating resources to the cognitive evaluation of individuals with reduced working capacity or sudden unemployment, due to the potentially elevated risk for a neurodegenerative disease. Based on these studies, it is evident that neurodegenerative diseases start to affect the person's social life and cause, for example, unemployment already a decade before the first dementia‐related symptoms are recognized.

The present study showed that non‐marital status was significantly higher among patients with EOVaD, EOVaD, and EOAD. Compared to controls, widowed status was more common in the AD, VaD, and EOVaD groups. Previous studies from Sweden and Japan have reported that unmarried individuals, including those who are widowed, divorced, or single, have an increased risk of both EOD and LOD compared to those who are married.^1^, ^9^ Similarly, a study from the United States found that remaining unmarried in midlife and later life may be a risk factor for dementia.^7^ Our study also found that single marital status was significantly more prevalent among EOAD patients than in the control group, particularly in females. On the other hand, FTD patients, regardless of sex or age group, did not differ from their controls regarding the marital status variables in the time period of 0 to 10 years before dementia. These findings together suggest and highlight disease‐specific patterns in the observed differences in marital status prior to dementia.

Some previous studies have shown that divorced older adults have the highest risk of dementia among the non‐married dementia population.^1^, ^7^ In the present study, more divorces compared to controls were mostly observed 5 years before the diagnosis in the FTD group, but the difference was not statistically significant. Thus, our results do not support the idea that specifically divorces would represent a significant risk or protective factor or early social consequence of EOD.

We found that individuals with VaD and AD + VaD had lower level of education. On the other hand, lower level of education, in general, associates with increased risk related to arterial diseases,^19^, ^20^ which may explain the observed results. Previous studies have also estimated that older subjects who had fewer years of education were at a greater risk of developing AD.^21^, ^22^ High education attainment has consistently been associated with a lower risk of cognitive impairment and dementia via increased cognitive reserve,^8^ but these previous studies have been somewhat limited compared to the size of our cohort and systematic data available from all individuals.

The major strengths in the present study are as follows. The Finnish health register system allowed us to collect essential data of sociodemographic factors that could not have been obtained otherwise. The long follow‐up time in the population and longitudinal health registers provided a great opportunity to study the long‐term association between the sociodemographic factors and different dementia subtypes. Also, each of the diagnoses were retrospectively manually reviewed and re‐assessed by a neurologist, entailing high specificity. The assessment took into account also all the follow‐up data. In addition, all the data from primary health care and regional hospitals are electronically available within the same database and retrievable according to the individual social security number of the patient. This increases the certainty of the diagnoses compared to a purely data‐based study. Moreover, public hospitals in Finland are known for their high quality in diagnosing neurodegenerative diseases and the diagnoses in more than half of the AD patients are validated using standard biofluid AD biomarker studies. The regionally selected age‐stratified control population without a neurodegenerative disease is also an important strength of this study. Due to the Finnish health‐care system, our cohort comprehensively represents the whole population in the two provinces, as all citizens regardless of their socioeconomic status are diagnosed using similar procedures in the health‐care system if a neurodegenerative disease is suspected. The study period of 12 years increases the validity of the data by decreasing the effect of annual changes in the diagnostics.

Our study also has some limitations. The study is retrospective and observational and does not attempt to infer causality. In addition, the reason for the unemployment status in this study could not be fully determined. Furthermore, this study did not focus on examining the interactions between different sociodemographic factors. Finally, the data used in this study might not be generalizable to other societies outside the Nordic countries having different health‐care systems.

In conclusion, our study demonstrates that differences in specific sociodemographic factors (“social markers”) may precede and indicate the onset of common dementias as early as 10 years before a clinical diagnosis. This is the first study showing a strong long‐term association between employment status and dementia. Employment status was lower at least 10 years before the diagnosis of the FTD, AD, VaD, and AD + VaD compared to controls, with the difference being most evident in EOFTD. Our study provides evidence that widowhood is associated especially with AD, VaD, and AD + VaD, but not with FTD. Lower level of education was found to associate only with VaD and AD + VaD. Furthermore, the study highlights that specific sociodemographic factors, especially lower employment status, are linked to the prodromal phases of common dementias. This underlines the need for earlier diagnoses, better understanding of prediagnostic features, and the development of appropriate pathways for emerging disease‐modifying treatments as well as social support for patients and their families. Subclinical changes in brain health are thought to begin more than two decades before the onset of overt dementia symptoms.^23^ We believe that these specific social markers could be part of risk prediction models to predict the clinical progression from subjective cognitive decline to objective cognitive impairment. Identifying individuals at risk of clinical progression can help target health‐care resources to those who appear normal but are at high risk of developing dementia.^24^, ^25^

## CONFLICT OF INTEREST STATEMENT

The funders of the study had no role in study design, data collection, data analysis, data interpretation, or writing of the report. E. Solje has served on the advisory board of Novartis, Eisai, Lilly, and Roche; served as a consultant for Novo Nordisk, BioArctic, Eisai, Lilly, and Roche; received honoraria for lectures from Lundbeck, BioArctic, Lilly, and Roche; received support for attending a conference from Lilly; and received payment for expert testimony from BioArctic. J. Krüger has served on the advisory board of Novartis, Eisai, Lilly, Nutricia, and Roche; received honoraria from lectures from BioArctic and Lilly; and received support for congress participation from Merck and Lilly. S. Heikkinen has received lecture fees from Finnish Brain Association and Nordic Infucare. A. Kivisild has received travel support for congress participation from Merck and Infucare. Other authors report no conflicts of interests. Any author disclosures are available in the supporting information. This study is part of DEGE‐RWD project, funded by Roche Oy. Other funding: A.H. (Sigrid Jusélius Foundation), S.H. (Finnish Brain Foundation, The Finnish Parkinson Foundation), K.K. (Finnish Brain Foundation, Uulo Arhio Foundation, The Finnish Medical Foundation, The Finnish Cultural Foundation), A.K. (The State Research Funding, Finnish Brain Foundation), J.K. (Wihuri Foundation, The State Research Funding), E.S. (Wihuri Foundation, Sigrid Jusélius Foundation, The State Research Funding), H.S. (Finnish Brain Foundation).

## CONSENT STATEMENT

Informed consent was not necessary.

## Supporting information



Supporting Information
